# Innate Immune Dysfunctions in Aged Mice Facilitate the Systemic Dissemination of Methicillin-Resistant *S. aureus*

**DOI:** 10.1371/journal.pone.0041454

**Published:** 2012-07-26

**Authors:** Ching Wen Tseng, Pierre A. Kyme, Andrea Arruda, V. Krishnan Ramanujan, Wafa Tawackoli, George Y. Liu

**Affiliations:** 1 Division of Pediatric Infectious Diseases, Cedars-Sinai Medical Center, Los Angeles, California, United States of America; 2 Department of Pediatrics, David Geffen School of Medicine, University of California Los Angeles, Los Angeles, California, United States of America; 3 The Immunobiology Research Institute, Cedars-Sinai Medical Center, Los Angeles, California, United States of America; 4 Department of Surgery & Department of Biomedical Sciences, Cedars-Sinai Medical Center, Los Angeles, California, United States of America; University of Liverpool, United Kingdom

## Abstract

Elderly humans show increased susceptibility to invasive staphylococcal disease after skin and soft tissue infection. However, it is not understood how host immunity changes with aging, and how that predisposes to invasive disease. In a model of severe skin infection, we showed that aged mice (16- to 20-month-old) exhibit dramatic bacterial dissemination compared with young adult mice (2-month-old). Bacterial dissemination was associated with significant reductions of CXCL1 (KC), polymorphonuclear cells (PMNs), and extracellular DNA traps (NETs) at the infection site. PMNs and primary skin fibroblasts isolated from aged mice showed decreased secretion of CXCL2 (MIP-2) and KC in response to MRSA, and *in vitro* analyses of mitochondrial functions revealed that the mitochondrial electron transport chain complex I plays a significant role in induction of chemokines in the cells isolated from young but not old mice. Additionally, PMNs isolated from aged mice have reduced ability to form NETs and to kill MRSA. Expression of nuclease by *S. aureus* led to increased bacterial systemic dissemination in young but not old mice, suggesting that defective NETs formation in elderly mice permitted nuclease and non-nuclease expressing *S. aureus* to disseminate equally well. Overall, these findings suggest that gross impairment of both skin barrier function and innate immunity contributes to the propensity for MRSA to disseminate in aged mice. Furthermore, the study indicates that contribution of bacterial factors to pathogenicity may vary with host age.

## Introduction

*Staphylococcus aureus* is an important pathogen that causes a wide variety of infections, ranging from skin and soft tissue infections to severe invasive diseases, such as sepsis and endocarditis. Approximately 35% of skin and soft tissue infection cases occur in people over 65 years of age, and 25% of these subjects later present with invasive staphylococcal diseases [1], [2], [3], [4]. Hence, for elderly people, skin and soft-tissue staphylococcal infections are an important risk factor for the development of serious invasive staphylococcal diseases. However, how old age contributes to invasive staphylococcal diseases is not fully understood.

Prior studies have demonstrated that immune cells from elderly subjects, compared to cells isolated from young subjects, exhibit reduced chemotaxis, phagocytosis, and respiratory burst activity in response to bacterial infection [5], [6], [7]. An *in vitro* study of murine peritoneal macrophages attributes the decreased level of inflammation in elderly to lower levels of Toll-like receptors (TLR) expression on cell surfaces [8], suggesting that innate immune activation decreases with aging. Furthermore, decline in cellular and inflammatory responses has been shown to be associated with dysfunction of mitochondria in cells isolated from aged hosts [9]. Therefore, decreased cellular functions and immune responses may result in ineffective clearance of bacteria and facilitate systemic infection.

To prevent the systemic spread of pathogens, polymorphonuclear cells (PMNs) form neutrophil extracellular DNA traps (NETs) containing DNA, histone, granule enzymes, and antimicrobial components [10], [11], [12]. NETs promote killing of microorganisms and trap microbes locally [13], thereby preventing dissemination of the pathogens. Several host factors have been shown to contribute to the formation of NETs, including TLR and interleukin (IL)-8 [11], [14]. Although NETs have been shown to interact with *S. aureus* and facilitate their killing [10], the effectiveness of NETs in aged mice against *S. aureus* has not been studied.

To investigate whether aging affects the outcome of MRSA infection, we utilized a murine skin infection model and studied host innate immune response to MRSA infection in young and aged mice. We showed that, compared to aged (16- to 22- month-old), mice young (2- month-old) mice exhibit reduced levels of MIP-2, KC, PMN recruitment, and NETs formation, and enhanced systemic dissemination of MRSA in response to infection. Furthermore, we demonstrated that mitochondrial electron transporter chain complex I could have a differential effect on chemokine response to *S. aureus* infection in young and aged mice. The importance of defective NETs in promoting systemic dissemination in elderly mice is suggested by the ability of *S. aureus* nuclease to support *S. aureus* dissemination in young but not old mice.

## Results

### Aged Mice are More Susceptible to MRSA Dissemination and Persistence Compared to Young Mice

**Figure 1:**
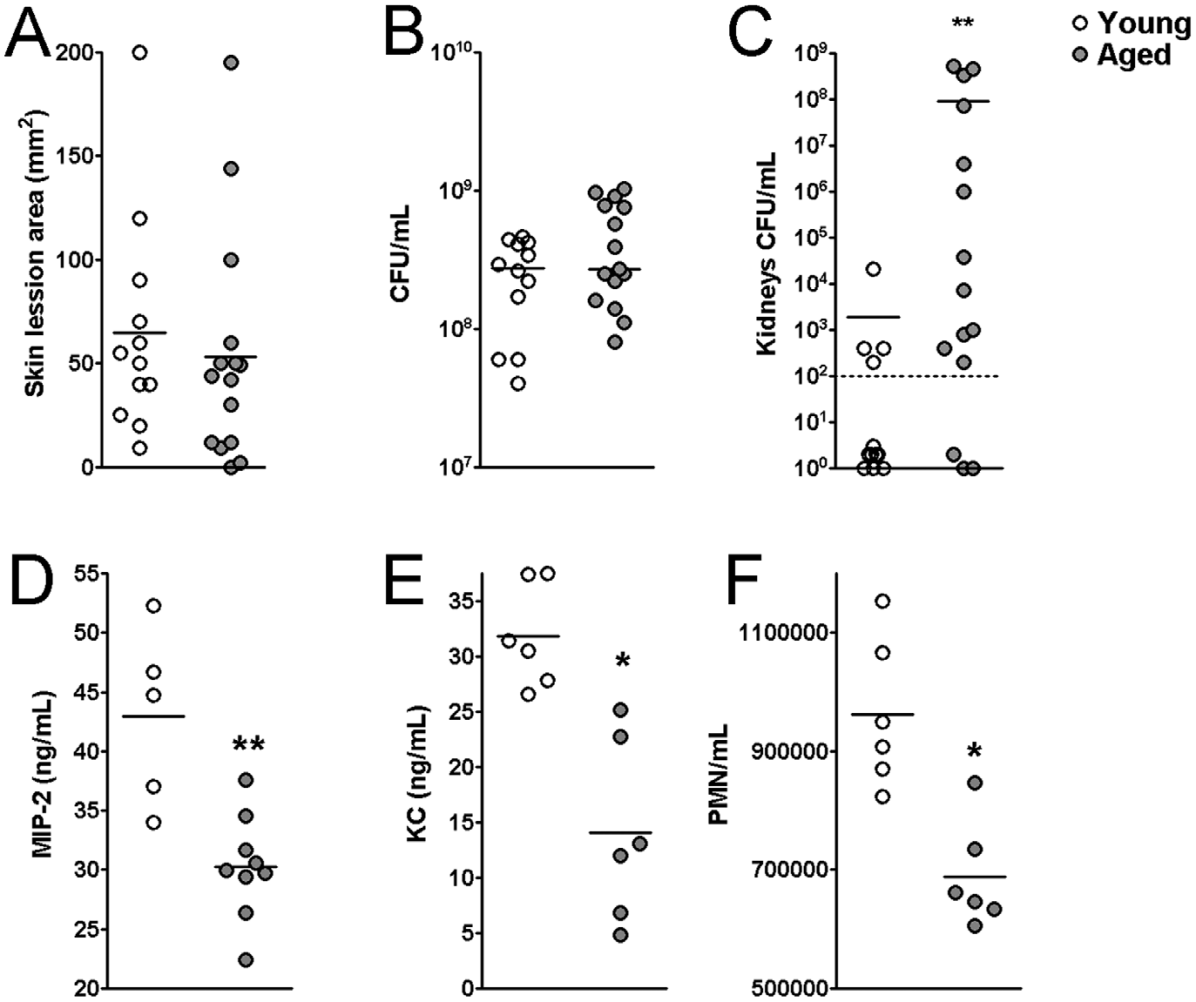
MRSA infection induces lower KC and PMNs at the infection site in aged mice. Age- and gender- matched mice were infected subcutaneously (s.c.) with 10^9^ CFUs of CST9. On day 3 post-infection, the mice were sacrificed, and (A) skin lesion size, (B) skin CFUs, and (C) kidney CFUs were determined. (D) MIP-2, (E) KC, and (F) PMNs from the lesions were quantitated at 3 hours post-infection. At least 3 mice were infected for each time point for IVIS analysis. At least 3 mice in each age group were infected for the chemokine, cytokine, and PMN analyses. *p<0.05, **p<0.01 for comparisons of young and aged mice.

Older people with MRSA skin and soft tissue infection have an increased risk of developing invasive MRSA disease [3], suggesting that age is a significant contributing factor for invasive staphylococcal infection. Macrophages isolated from aged mice show reduced TLR expression on cell surfaces, which may contribute to reduced inflammation in elderly individuals [8]. Because of these findings, we hypothesized that reduced inflammatory response in aged mice could lead to more invasive MRSA infection. In a murine skin infection model, we observed no significant difference of skin lesion size and bacterial burden between young and aged mice on day 3 post-infection (**Fig. 1A and B**). However, older mice showed a dramatic increase in systemic bacterial burden compared to young mice (**Fig. 1C**). At the site of bacterial inoculation, approximately 1.5 fold lower levels of MIP-2 and 2-fold lower KC were observed at 3 hours post-infection in aged mice compared with young mice (**Fig. 1D and 1E**). Because of the lower chemokine levels (MIP-2 and KC), we investigated PMN recruitment to the infection site using a MPO assay. As shown in **Fig. 1F**, significant fewer PMNs were found at the infection site in the aged mice at 3 hours post-infection. These results suggest that impaired chemokine response in aged hosts contributes to the defective immune cell recruitment.

Using a Xenogen imaging system and an isogenic MRSA harboring Tn4001::*luxABCDE* Km^R^ [15], we examined the invasiveness and persistence of MRSA infection over a period of 14 days in both young and aged mice (**File S1** and **Fig. S1A**). On day 1 post-infection, dense bioluminescence was observed at the infection site in both young and aged mice. However, in aged mice, diffuse bioluminescence was also notable throughout deeper tissues. By day 14 post-infection, the young mice exhibited dense bioluminescence only at the skin infection site, whereas the aged mice continued to display both skin and systemic bioluminescence. Enumeration of CFU showed a significantly higher bacterial burden in the skin and kidneys of aged mice compared to young mice (**File S1**, **Fig. S1B and S1C**). Taken together, these results suggest that the aged mice may develop more invasive and persistent MRSA infection after skin infection.

### PMNs Isolated from Aged Mice have Reduced Chemokine Responses to MRSA

**Figure 2:**
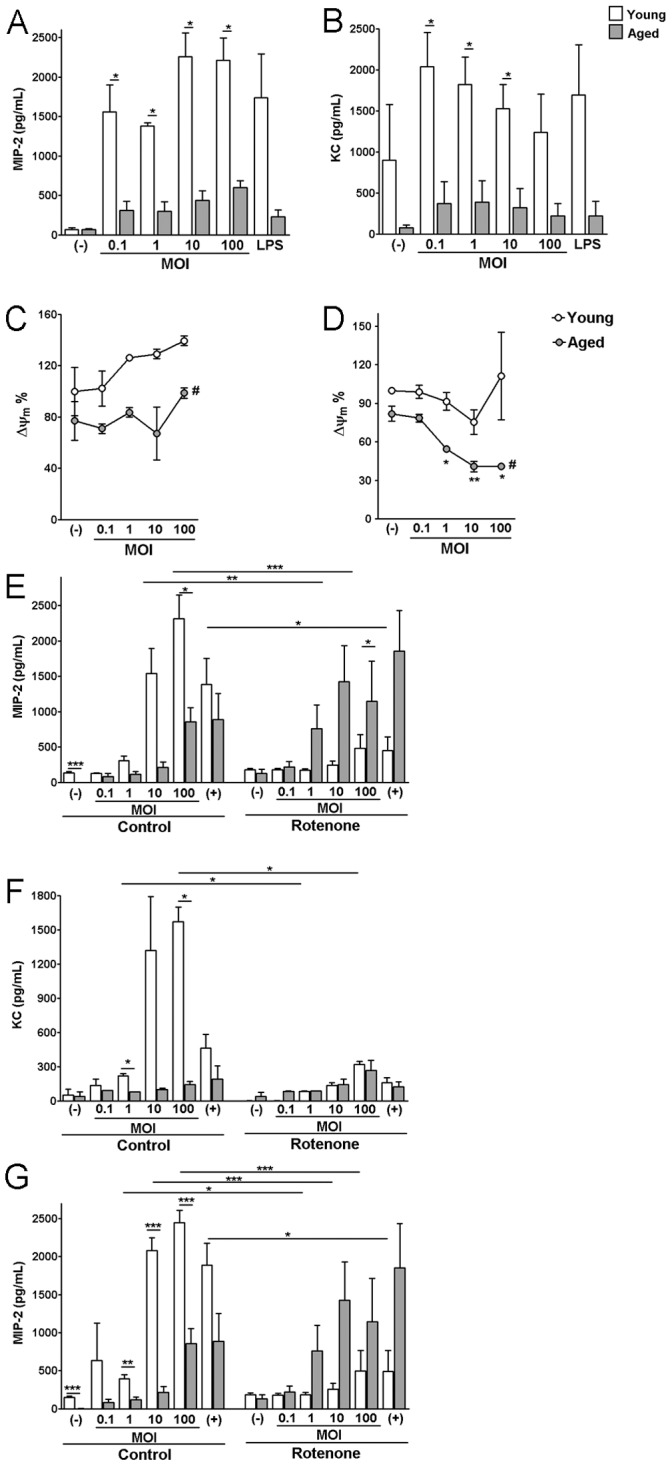
Mitochondrial electron transporter chain complex I plays an important role in the induction of MIP-2 in primary cells isolated from young but not old mice. (A) PMN KC at 18 h post-infection; (B) PMN MIP-2 at 18 h post-infection. (C) PMN membrane potential at 3 hours post-infection. (D) PMN membrane potential at 18 hours post-infection. The fluorescence of uninfected PMNs isolated from young mice is arbitrary set as 100%. (E–G) Primary cells were infected for 18 h with MRSA in the presence or absence of rotenone, and culture supernatants were collected for chemokine analyses. (E) PMN MIP-2, (F) PMN KC, and (G) skin fibroblast MIP-2. *p<0.05, **p<0.01, ***p<0.005. #*p*<0.05 for membrane potentials of young versus aged PMNs after infection with MRSA across a range of MOIs.

Next, we asked whether immune cells or stromal cells play a role in the reduced chemokine responses. Since PMNs contribute to the induction of inflammation at the infection site [16], PMNs and primary skin fibroblasts were isolated from different age groups of mice. PMNs isolated from young and aged mice were infected with MRSA across a range of MOIs. At 18 h post inoculation, PMNs from aged mice showed lower levels of MIP-2 (**Fig. 2A**) and KC (**Fig. 2B**), compared to PMNs from young mice. Similar MIP-2 response to MRSA was observed in peritoneal macrophages isolated from young and aged mice (**File S1** and **Fig.** **S2A**). Skin fibroblasts isolated from aged mice also revealed a reduced MIP-2 response compared to fibroblasts isolated from young mice (**File S1** and **Fig.** **S2B**). Additionally, LPS stimulation revealed reduced responses in aged hosts, which is in agreement with previously published work that cells isolated from aged hosts have reduced immune responses to LPS resulting from reduced levels of TLR expression on cell surfaces [8]. Taken together, we showed that select primary cells from the aged mice secrete lower levels of chemokines when challenged with MRSA.

### Mitochondrial Electron Chain Transport Complex I (Complex I) Plays a Significant Role in Host Chemokine Responses to MRSA in Young Mice

Mitochondrial activities are critical to many cellular functions and to host inflammatory pathways activated in response to infection [17]. Mitochondrial functions decrease with advanced age and contribute to cellular dysfunctions observed in elderly hosts [18]. Therefore, dysfunction of mitochondria could be intrinsically linked to the decline of chemokine response in aged mice. To test this hypothesis, we first examined whether PMNs isolated from young and aged mice have different levels of mitochondrial membrane potential using tetramethylrhodamine methyl ester (TMRM). TMRM is a mitochondrial membrane potential sensitive dye which is used to track the mitochondrial membrane potential [19]. As shown in **Fig. 2C and 2D**, at 3 and 18 hours post-infection, PMNs isolated from young mice show significantly higher levels of fluorescence intensity than PMNs from aged mice, suggesting that greater depolarization occurred in the mitochondrial membrane of PMNs from the aged animals.

Since the function of complex I is primarily associated with the mitochondrial membrane potential and is affected by aging [20], we next asked whether inhibition of complex I using rotenone (complex I specific blocker) has an impact on chemokine secretion by PMNs. Because high concentrations of rotenone treatment induces apoptosis, a dose titration of rotenone was performed to determine the concentration required to collapse the mitochondrial membrane potential without inducing apoptosis of the cells (data not shown). As shown in **Fig. 2E and 2F**, control PMNs isolated from aged mice showed lower levels of MIP-2 and KC in the culture supernatant at 18 hours post-infection, compared to PMNs from young mice. When treated with 20 nM rotenone, PMNs from young mice revealed significant reductions of MIP-2 and KC in the culture supernatant. By contrast, rotenone treatment had a minimal effect on PMNs from aged mice. Under certain conditions, rotenone treatment resulted in increased levels of MIP-2 in the aged cells, suggesting mitochondrial complex I may have differential effect on inflammatory responses in young and aged hosts. The underlying cause of this differential effect is unclear. Similar assays were also performed using skin fibroblasts isolated from young and aged mice. Consistent with the results obtained with PMNs, skin fibroblasts from aged animals secrete a lower level of MIP-2 (**Fig. 2G** **)** and KC (**File S1** and **Fig. S3A and S3B**) when infected with MRSA, and were less responsive to rotenone repression of Complex I functions, compared to skin fibroblasts from young mice. These results are consistent with a prior report that cells isolated from aged animals are less responsive to rotenone treatment [21]. However, no significant difference in KC was observed when infected skin fibroblasts were treated with antimycin A (Complex III inhibitor) or oligomycin (Complex V inhibitor) (**File S1** and **Fig. S3**). Overall, these results suggest that functional complex I plays a significant role in KC and MIP-2 responses to MRSA infection in young but not aged mice.

### PMNs Isolated from Aged Mice have Reduced Anti-MRSA Activity and Reduced NETs Formation

**Figure 3:**
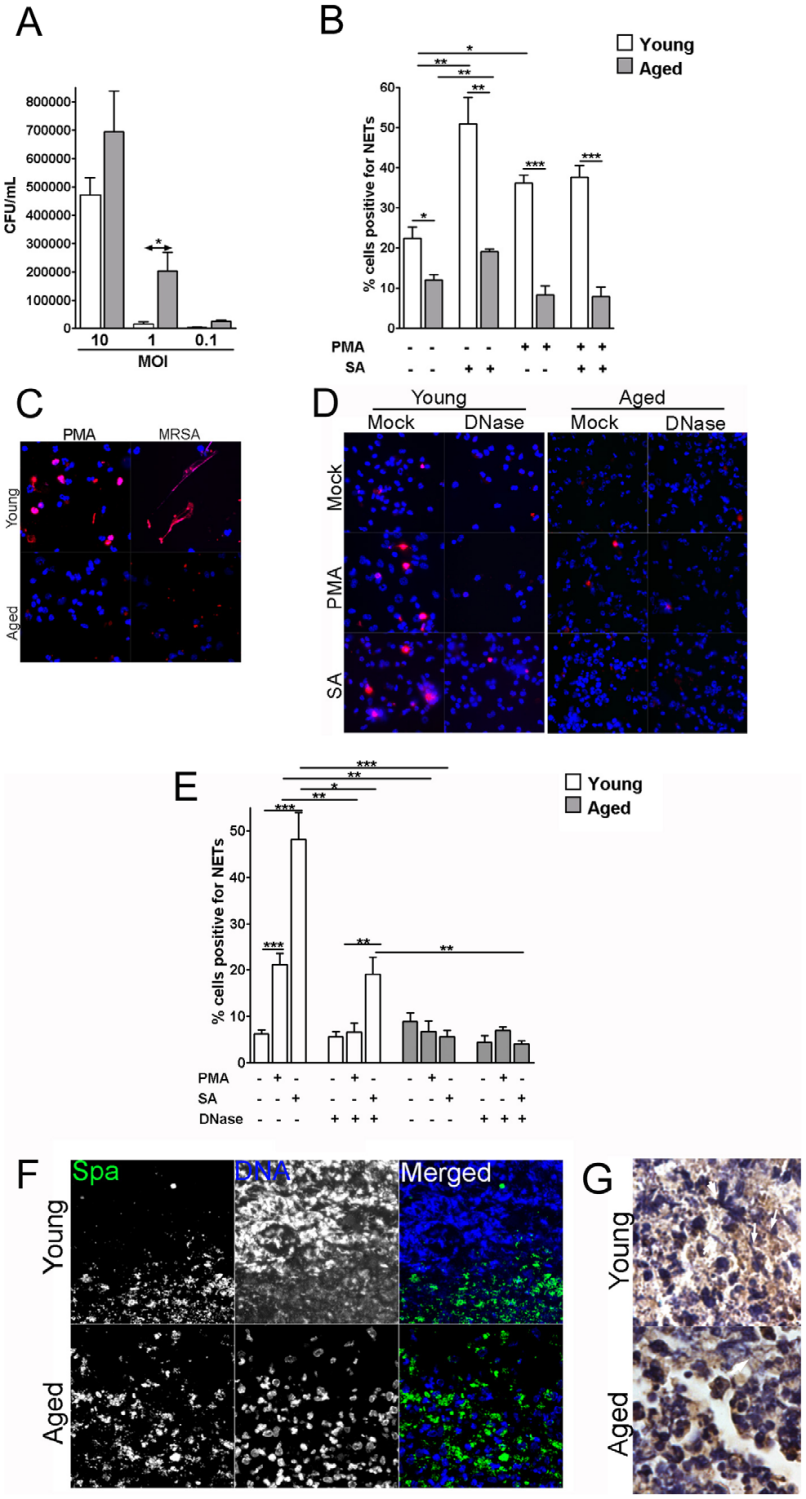
Aged mice show defective NETs formation in response to MRSA. (A) PMNs show reduced bactericidal activities against MRSA at 1 hours post infection, *in vitro* (n = 3). (B–C) PMNs from young and aged mice were incubated with or without 20 nM PMA for 1 h, and then with either MRSA at MOI of 10 or PBS for another 2 h. (B) Quantitative analysis of percentage of cells that is NETs positive (n = 3). (C) Representative image of *in vitro* NETs formation. Cells were stained for histone (Red) and DNA (Blue). (D–E) PMNs from young and aged mice were incubated with 20 nM PMA or MRSA at MOI of 10 with or without 100 ng of DNase I for 2 h. (D) Representative images of *in vitro* NETs formation with or without DNase treatment. Cells were stained for histone (Red) and DNA (Blue). (E) Quantitative analysis of percentage of cells that are NETs positive (n = 3). (F) NETs formation within skin lesions on day 3 post-infection. Infected tissues were stained for staphylococcal protein A (Spa) (green) and DNA (blue) (400×). (G) Immunochemical stain of elastase within skin lesions on day 3 post-infection. Infected tissues were stained for elastase and counter stained with hematoxylin (1000×). *p<0.05, **p<0.01, ***p<0.005.

Prior studies have shown that PMNs isolated from aged hosts have reduced ability to generate oxygen metabolite and to phagocytose *S. aureus*, compared with young mice [6]. To examine whether PMNs from aged mice have reduced ability to kill MRSA, an *in vitro* PMN killing assay was performed. As shown in **Fig. 3A**, PMNs from aged mice were ineffective at clearing MRSA in the suspension, consistent with a previous report that PMNs from elderly people have reduced ability to kill bacteria [22].

During acute inflammation, formation of neutrophil extracellular traps (NETs) by PMNs is a critical host strategy that kills invading organisms and prevents them from systemic dissemination [10]. To examine the possibility that young and aged mice have different ability to form NETs, PMNs were isolated from young and aged mice and assessed for NETs formation. As shown in **Fig. 3B and C**, following treatment with PMA or infection with MRSA, PMNs from young mice exhibited ∼2.5 fold higher concentration of NETs compared to PMNs from aged mice. To correlate to *in vivo* findings, infected tissues procured from young and aged mice were stained for DNA. DNase treatment has been shown to eliminate NETs formation [23]. Therefore, to examine whether DNase treatment would abrogate the NETs *in vitro*, PMNs were isolated from mice of different age groups and treated with or without DNase I during induction of NETs by PMA and *S. aureus*. As shown in **Fig. 3D**, reduced amount of the extracellular DNA fibers were observed in the DNase treated samples. Additionally, reduced numbers of PMNs were positive for the NETs formation with DNase treatment for PMNs derived from young mice. This is in contrast to PMNs isolated from aged hosts for which no significant difference in NETs was observed with or without DNase treatment (**Fig. 3E**). *In vivo* (**Fig. 3F**), infected skin tissues from young mice revealed meshed networks of extracellular DNA stain at the edge of the infection site, with very few bacteria notable beyond the extracellular DNA meshes. By contrast, this extracellular DNA network was not observed in the aged mice. To examine whether the increased levels of the extracellular DNA networks in the infected tissue of young mice are NETs, we stained the infected tissue collected from both young and aged mice with elastase (**Fig. 3G**). In the tissue section of the infected young mice, increased levels of cellular debris, extracellular elastase, and DNA fibers positive of elastase were observed, which is in contrast to tissue sections collected from infected aged mice. Furthermore, we examined whether the increased levels of DNA fibers in young could be degraded by DNase. To avoid destruction of DNA fibers by the nuclease expressed by *S. aureus*, young mice were infected with 10^9^ CFU of nuclease-negative (Nuc^-^) *S. aureus* with or without daily treatment with 1 µg of DNase I. On day 3 post-infection, the mice that received the DNase I treatment showed reduced amount of DNA fiber at the infection site while the mice that received Nuc^-^ *S. aureus* infection had increased amount of DNA fibers at the infection site (**Fig. S4**). Similar reductions in DNA fibers were observed in all DNase I treated mice. Thus, the impairment of PMN recruitment and reduced NETs formation in aged hosts, among others, may lead to more invasive MRSA infection in aged hosts.

### Nuclease is a Virulence Factor in Young but not Aged Mice

**Figure 4:**
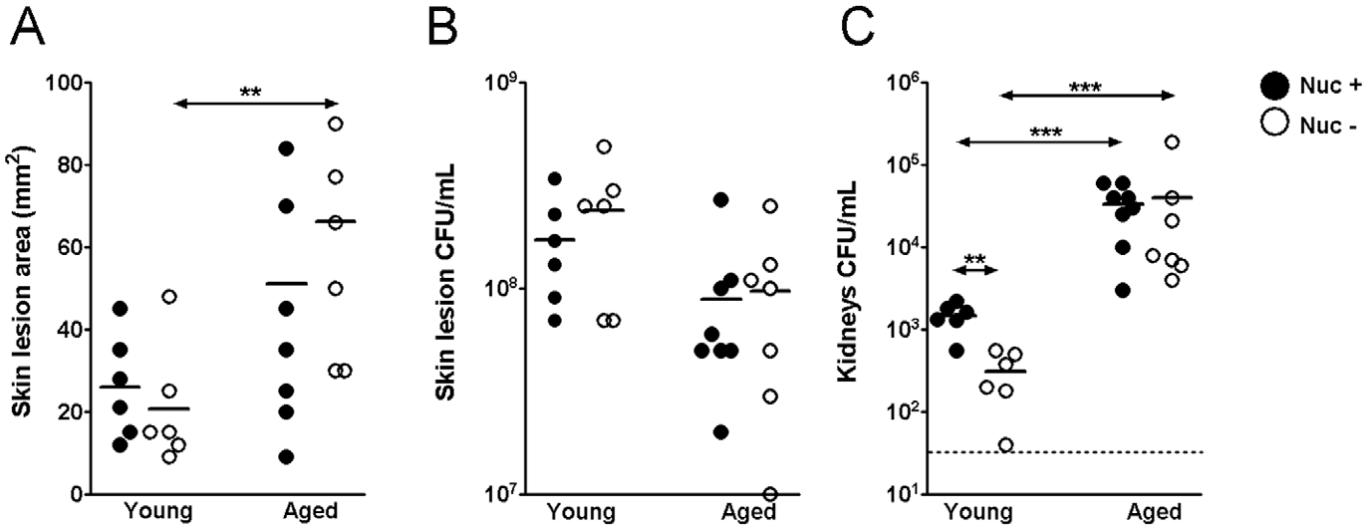
Expression of *S. aureus* Nuclease increases *S. aureus* burden in the kidneys of young, but not aged mice. Gender- and age- matched mice were infected subcutaneously (s.c.) with 10^9^ CFUs of a nuclease expressing *S. aureus* (UAMS 1552) (Nuc+) and an isogenic nuclease non-expressing *S. aureus* (UAMS 1472) (Nuc–). On day 3 post-infection, the mice were sacrificed, and (A) skin lesion size, (B) skin CFUs, and (C) kidney CFUs were measured. *p<0.05.

Prior studies have shown that nuclease expression by pathogens leads to destruction of NETs and facilitates systemic dissemination of the bacteria [13], [24], [25], [26]. We have shown in this study that the increased NETs formation occurs at the infection site of young mice (**Fig. 3G**). To test the hypothesis that deficiency of NETs formation in aged hosts leads to systemic dissemination of *S. aureus*, we infected young and aged mice with an *S. aureus* nuclease knockout and its isogenic nuclease expressing strain. As shown in **Fig. 4A**, nuclease expression did not have a significant effect on skin lesion size in each age group. Consistent with previous skin CFU findings from the MRSA infection (**Fig. 1B**), young and old mice infected with either strain of *S. aureus* show no difference in skin CFUs (**Fig. 4B**). However, nuclease expression in *S. aureus* contributed to an approximately 5 fold higher bacterial burden in the kidney in young mice but not in old mice (**Fig. 4C**). Additionally, young and old mice show an approximately 100 fold difference in kidney CFUs after infection with either strain of *S. aureus*. These results demonstrate that nuclease is important for the dissemination of *S. aureus* in young mice but not in aged host. Furthermore, the data suggest that defective NETs formation contribute, at least in part, to the increased dissemination of *S. aureus* in aged hosts.

## Discussion

With an aging global population, there is an increasing need to understand why elderly individuals are more susceptible to infection. Age-dependent immune changes are likely to contribute to the increased incidence of severe infections [2], and based on epidemiologic studies, the elderly population is more prone to invasive MRSA disease [3]. Aging impacts a number of innate immune functions, impairing the host’s ability to clear invading pathogens [27]. Epidemiologic studies reported an increased incidence of soft-tissue infections in the elderly population [28], and increased susceptibility to invasive disease after a cutaneous infection [4]. Results from our murine model closely mimic the human clinical features in that we do not find a difference in skin lesion size, but observe a dramatic difference in MRSA dissemination into the bloodstream between young and aged mice.

Based on our finding that PMNs from aged mice are impaired in their ability to kill MRSA, we initially hypothesized that aged mice would exhibit a high CFU burden in the subcutaneous tissues, and this would lead to bacterial dissemination into deep tissues by sheer spill over effect. Though a dramatic difference in dissemination of MRSA was observed, unexpectedly, there was no difference in bacterial burden between young and aged animals under the skin. We interpreted these data as evidence of a prominent defect in skin barrier function in aged mice.

Current understanding of mechanisms that limit bacterial dissemination is incomplete. However, a critical factor that has been implicated is the formation of NETs by PMNs which facilitate trapping and killing of pathogens during infection [10], [25]. Published studies have shown that pathogens that have an ability to evade NETs entrapment have a greater predilection to disseminate [26], [29]. We showed in aged mice that both PMN recruitment and NETs formation are severely impaired. Reduced PMNs could be explained at the cellular level by the inability of primary cells from aged mice to produce chemokines (KC and MIP-2) to recruit PMNs. Reduced PMNs in turn would lead to reduced NETs formation. Additionally, we demonstrated that aged PMNs have an inherent defect in NETs formation. Previous studies have shown that nuclease expression allows pathogens to escape NETs and promote systemic dissemination of the pathogen [24], [25], [26]. Consistent with the hypothesis that deficiency of NETs in aged mice promotes systemic dissemination of MRSA, we showed that nuclease expression by *S. aureus* leads to enhanced bacterial dissemination in young but not old mice. Taken together, the result suggests that defective NETs formation plays a role in the increased *S. aureus* dissemination in aged mice, but it does not exclude the possibility that other factors, such as defective skin structural barrier functions, are also involved in the increased invasiveness of *S. aureus* in aged hosts. Further investigations are necessary to tease out the detailed mechanisms.

The finding that nuclease promotes bacterial growth in young but not old mice point to a concept that virulence could be age specific. It is well appreciated from studies of mutant mice or mice of different backgrounds that bacterial virulence is sometimes dependent on host expression of factors that interact with the bacterial factor. For example, pathogenicity associated with LPS requires host expression of TLR4 [30]. Likewise, the concept that virulence is frequently tissue specific is also important because it informs on the effectiveness of anti-virulence strategies including vaccine development. However, the effect of host age on virulence of microbial factors is not well studied. Our demonstration that virulence is age specific points to the role of host immunity as a critical determinant of whether a virulence factor induces pathology. This concept could be important in the context that vaccines against specific virulence determinants could be effective against one age group but not another.

While many immune defects (e.g. phagocytosis, reactive oxygen section) are associated with aging, the fundamental age-dependent cellular changes that are responsible for these defects are not known. However, it is well appreciated that cells from aged individuals have altered mitochondrial functions which could impact their ability to respond to external stimuli, including pathogens. In our model, we showed that both skin fibroblasts and PMNs isolated from aged mice have reduced ability to secrete chemokines in response to MRSA, compared to young mice. Blocking of the mitochondrial electron chain complex I, but not complexes III or V, revealed a significant contribution of complex I function to chemokine responses in cells derived from young mice but not aged mice. Interestingly, blocking of mitochondrial complexes did not have the same effect on NETs formation (unpublished results). Therefore, age-dependent changes in mitochondrial functions could differentially account for changes in immune functions between young and older hosts. The exact role of mitochondrial function in *S. aureus* induced chemokine responses is not clear. Complex I function has been shown to play a role in lipopolysaccharide (LPS) induced inflammatory responses by modulating NF-κB activation [17], suggesting that TLR4-dependent immune activation pathway is dependent on the mitochondrial function. Pertinently, Sweeney *et. al*. reported that the up-regulation of mitochondrial biogenesis transcription factors in *S. aureus* sepsis is TLR2- and TLR4-dependent, which further suggests an interplay between mitochondrial functions and TLR-dependent innate immune activation [31]. Additional studies are required to elucidate how mitochondrial functions modulate *S. aureus-*induced inflammation in our model.

Overall, we have generated a murine model of MRSA infection in aged mice that captures the hallmark clinical finding that elderly individuals are more susceptible to invasive disease after skin infection, which may result from the grossly leaky barrier function and declined innate immunity in the aged mice. We have demonstrated defective chemokine response, PMN recruitment, NETs formation, and bacterial clearance in the aged mice that could explain these findings. Furthermore, we have shown that deficiency of NETs formation likely plays a role in promoting the dissemination of MRSA in aged hosts. To the best of our knowledge, the defective NETs formation in aged mice has not been described previously. Further experiments will be required to address the detailed mechanism of NETs dysfunctions in aged hosts, which could lead to specific immune boosting strategies.

## Materials and Methods

### Bacterial Strains and Growth Conditions

A clinical MRSA isolate from a skin infection (CST9) was used for this study. Nuclease positive *S. aureus* strain (UAMS 1552) and Nuclease negative isogenic strain (UAMS 1471) were generous gifts from Dr. Mark Smeltzer [32]. MRSA strains were routinely cultured on sheep blood agar plates, and colonies with comparable hemolytic phenotype were used for each experiment. Bacteria were also cultured in Todd-Hewitt broth at 37°C with shaking at 250 rpm.

### Murine Skin Infection Model

Two- and 6-month-old male and female CD1 mice were purchased from Charles River Laboratories. Six-month-old mice were maintained in the animal facility in Cedars-Sinai Medical Center until they reached 16- to 22-month-old. Infection of mice was performed using previously established protocols [33]. Briefly, an overnight bacterial culture was diluted 1∶200 in pre-warmed media and incubated at 37°C with shaking at 250 rpm until an A_540_ reached ∼2.5. Bacteria were harvested by centrifugation at 4000 rpm for 10 min at 4°C, and then washed twice with equal volume of Dulbecco’s PBS (DPBS) (Mediatech). The bacteria were resuspended in DPBS to a concentration of approximately 10^10^ CFU/mL. One hundred microliters of suspension was injected subcutaneously in each flank. Injections were performed with careful visualization of the needle to assure that the injection site was not intramuscular.

All animal experiments were approved by the Cedars-Sinai Committee on the Use and Care of Animals and performed using accepted veterinary standards.

### Determination of Lesion Size and Tissue Bacterial CFUs

Following euthanization, lesions were measured, then were excised and homogenized in 1 mL of DPBS for CFUs determination following previously established protocols [33]. Lesions defined by darkened area of necrosis were measured using previously established methods [34].

### Immunofluorescent Assays (IFA) and Immunochemical Analysis

Infected tissues were excised and fixed in 10% formalin (Medical Chemical Corporation) overnight. Paraffin embedding was performed by the Department of Pathology at Cedars-Sinai Medical Center. Tissue sections were deparaffinized and blocked with PBS with 0.05% Tween 20 (ISC Biosciences) and 0.5% BSA (blocking buffer) for 1 hour at room temperature. For *in vitro* assays, tissue samples were fixed with 10% formalin at room temperature for 30 min. The samples were blocked with PBS-0.05% Triton-X 100 and 0.5% BSA at room temperature for 1 hour. After incubation of samples with rabbit anti-staphylococcal protein A antibody (Sigma) at room temperature for 1 hour, slides were washed for 5 min with PBS 3 times. Then, the slides were incubated with corresponding FITC- conjugated secondary antibody (Jackson ImmunoResearch Laboratory), and hoechst (at a final concentration of 1 µg/mL) in PBS-0.5% BSA. After a final wash, the tissue sections were mounted with Prolong AntiFade (Invitrogen). The stained slides were examined using an Olympus BX51 fluorescent microscope.

For immunochemical analysis of elastase, the tissue sections were deparaffinized and processed for antigen retrieval using 10 mM sodium citrate buffer (pH 6.0) at 95°C for 10 min. The tissue section slides were washed for 5 min with PBST 3 times, and then incubated with 1∶100 diluted rabbit anti-elastase antibody (Abcam) at room temperature for 2 hours. After 3 washes for 5 min with PBST, 100 µL of the broad spectrum antibody detection solution was added to each sample following the manufacture protocol (Invitrogen). The slides were visualized by DAB solution (BD Biosciences) and counter stained by hematoxylin. After mounting using histomount (Invitrogen), the stained slides were analyzed using a BX51 microscope.

### Enzyme Linked Immunosorbent Assay (ELISA), and Myeloperoxidase (MPO) Assay

Animals were euthanized at 3 and 72 h post-infection, and the skin and muscle lesions were homogenized in 1 mL of PBS-Triton X-100 (0.05%) with protease inhibitor cocktails (Roche). The homogenized suspension was centrifuged at 15,000×*g* for 10 min, and supernatants were collected and examined using ELISAs and MPO assay. Mouse IL-10, MIP-2, and KC (R & D Systems) specific ELISAs were performed according to the manufacturer’s instructions. MPO activity (Invitrogen) was determined according to the manufacturer’s instructions, and isolated murine PMNs were used as standard to convert the MPO activity to PMN number.

### *In vitro* Infection of Primary Cells

Macrophages, PMNs [35], [36], and skin fibroblasts [37] were isolated from mice of different age groups using modified established protocols. For PMN isolation, 4% thioglycollate was injected into the peritoneal cavity of mice. After 4 hours, 7 mL of DPBS was injected into the peritoneal cavity to collect peritoneal cells. Isolated PMNs were washed with DPBS twice, and red blood cells were lysed using ddH_2_O. The cells were collected using centrifugation at 500×*g* for 10 min at 4°C. Cell viability was determined using trypan blue assay. Skin biopsy samples were digested in a Petri dish with 0.25% of trypsin (Invitrogen) for approximately 1 hour. The dermis layer was removed and minced into small pieces using sterile razor blades. The minced sample was transferred into a 50 mL conical tube and 10 mL of 0.25% trypsin was added to the sample and incubated at 37°C for 1 hour with gentle agitation. After the incubation, the suspension was filtered using a 70 µm tissue strainer, and the cells were collected by centrifugation at 700×*g* for 10 min. The cells were washed twice with DMEM and cultured in 20% FBS-DMEM with 1% penicillin and streptomycin in a 5% CO_2_ incubator. Passages 4 to 7 of cultured skin fibroblasts were used for *in vitro* experiments.

To assess the impact of mitochondrial electron chain complexes on specific cellular functions, isolated cells were incubated with or without 20 nM of rotenone (Complex I specific inhibitor) for 18 hours in DMEM-5% FBS in a 37°C 5% CO_2_ incubator. For PMNs, after rotenone treatment, cells were collected by centrifugation at 700×*g* for 10 min and washed with HBSS twice. PMNs were stained with trypan blue to verify viability before plating and infection with MRSA. The PMNs were resuspended in DMEM with 5% FBS with or without 20 nM of rotenone, and 1×10^4^ viable cells were plated in each well of 96 well plates for infection. For skin fibroblast, media were removed and cells were washed twice with HBSS, and 400 µL of DMEM with 5% FBS with or without 20 nM of rotenone was added to each well. Diluted MRSA were added to each well to a final MOI of 0.1 to 100. To facilitate the interaction of cells and MRSA, the tissue culture plates containing isolated cells and MRSA were centrifuged at 700×*g* for 10 min at room temperature. After centrifugation, the cells were incubated at 37°C with 5% CO_2_ for 3 and 18 hours. After incubation, the culture supernatants were collected and assayed using ELISAs.

### Assay for Mitochondrial Membrane Potential

Mitochondrial membrane potential was measured using a previously established tetramethylrhodamine methyl ester (TMRM) method [19]. Briefly, PMNs isolated from young and aged mice were washed with DPBS three times before resuspended in DMEM-5% FBS. Ten thousand viable PMNs were plated in each well in a 96 well tissue culture plate. Cells were infected with MRSA at MOIs ranging from 0 to 100. At 3 and 18 hours post-infection, the cells were centrifuged at 700×*g* for 10 min, and culture supernatants were carefully removed. The cells were then resuspended in DMEM containing 20 nM TMRM. After incubation at 37°C with 5% CO_2_ for 20 min, fluorescence was measured using a microtiter plate reader at excitation 540 nm and emission 570 nm.

### NETs Formation

NETs were visualized using a modified established protocol [38]. One hundred thousand viable PMNs isolated from different age groups of mice were seeded in each well, in tissue culture plates. The cells were treated with or without 20 nM of phorbol myristate acetate (PMA) for 1 hour before infection. Diluted bacterial suspensions were added to each well to a final multiplicity of infection (MOI) of 10. After addition of the bacterial suspension, the tissue culture plates were centrifuged at 700×*g* for 10 min at room temperature to facilitate interaction of the cells and the bacteria. After centrifugation, the plates were incubated at 37°C with 5% CO_2_ for 2 hours, fixed with 4% formalin at room temperature for 30 min. For DNase I treatment, 10^5^ viable PMNs isolated from mice of different age groups were seeded in each well in tissue culture plates. The cells were treated with or without 100 ng of DNase I (Sigma) plus PMA (20 nM) or MRSA at MOI of 10. After addition of the stimuli, the tissue culture plates were centrifuged at 700×*g* for 10 min at room temperature, incubated at 37°C with 5% CO_2_ for 2 hours, and fixed with 4% formalin at room temperature for 30 min. The samples were blocked with PBS-0.05% Triton-X 100 and 1% BSA (blocking buffer) at room temperature for 1 hour. After incubation of samples with rabbit anti-histone H3 body (AbCam) in blocking buffer at room temperature for 1 hour, slides were washed for 5 min with PBS 3 times. Then, the slides were incubated with corresponding Texas Red-conjugated secondary antibody (Jackson ImmunoResearch Laboratory) and hoechst (at a final concentration of 1 µg/mL) in PBS-0.5% BSA. After a final wash, the tissue sections were mounted with Prolong AntiFade (Invitrogen). The stained slides were examined using an Olympus BX51 fluorescent microscope. The total number of PMNs and the number of NETs-positive PMNs were counted from five random fields of each sample. The percentage of NETs-positive cells was determined as the number of histone-positive cells divided by the total number of cells multiplied by 100%.

### Statistical Analysis

Data were analyzed using unpaired *t* – test unless otherwise indicated (Prism 4.03, Graphpad Software, Inc.). *p* values less than 0.05 were considered significant, and were noted in the figures. In bar graphs, results were presented as mean ± SEM (n≥3).

### Ethics Statement

This study was performed under strict accordance with the recommendations in the Guide for the Care and Use of Laboratory Animals. CSMC is accredited by the Association for Assessment and Accreditation of Laboratory Animal Care International (AAALAC), and in compliance with NIH guideline of laboratory animal care and use. The protocol was approved by the institutional animal use and care committee of the Cedars-Sinai Medical Center (IACUC 3402). All procedures were performed under isoflurane anesthesia, and all efforts were made to minimize suffering.

## Supporting Information

Figure S1 Examination of invasiveness and persistence of subcutaneously injected MRSA. Young and aged mice were infected s.c. with 10^9^ CFUs of CST9 and followed for 14 days. (A) Visualization of MRSA dissemination. CST9 harboring Tn4001::*luxABCDE* Km^R^ was used for infection of mice. Bioluminescence at the infection site and deep tissues was followed over a period of 14 days using an IVIS system. At least 3 mice in each age group were infected for each time point for IVIS analysis. b: back of mice; d: dorsa of mice. (B) Skin lesion CFU (n = 6); (C) CFU in Kidneys (n = 6).
(TIF)

Figure S2 Macrophages and skin fibroblasts isolated from aged mice show reduced MIP-2 response to MRSA infection. (A) Macrophage MIP-2 at 18 h post-infection; (B) skin fibroblast MIP-2 at 18 h post-infection. *p<0.05, compared between young and aged mice.
(TIF)

Figure S3 Mitochondrial electron chain complex III and V have limited effect on KC levels in skin fibroblasts isolated from both young and aged mice when responding to MRSA infection. (A) Young; (B) Aged.
(TIF)

Figure S4 *In vivo* DNase administration reduced DNA fibers at the site of infection. Young mice were infected with 10^9^ CFU of Nuc^-^ *S. aureus* and half were treated daily with 1 µg DNase I. On day 3 post-infection, the mice were sacrificed and the infected tissues were excised. After embedding, the tissue slices were analyzed for the presence of elastase by immunohistochemistry.
(TIF)

File S1 Supporting methods and materials.
(DOC)
