# Supplementary figures and images for: Innate Immune Dysfunctions in Aged Mice Facilitate the Systemic Dissemination of Methicillin-Resistant S. aureus
Source: PLoS One. 2012 Jul 26;7(7):e41454. doi: 10.1371/journal.pone.0041454 (PMC3406035; doi:10.1371/journal.pone.0041454)

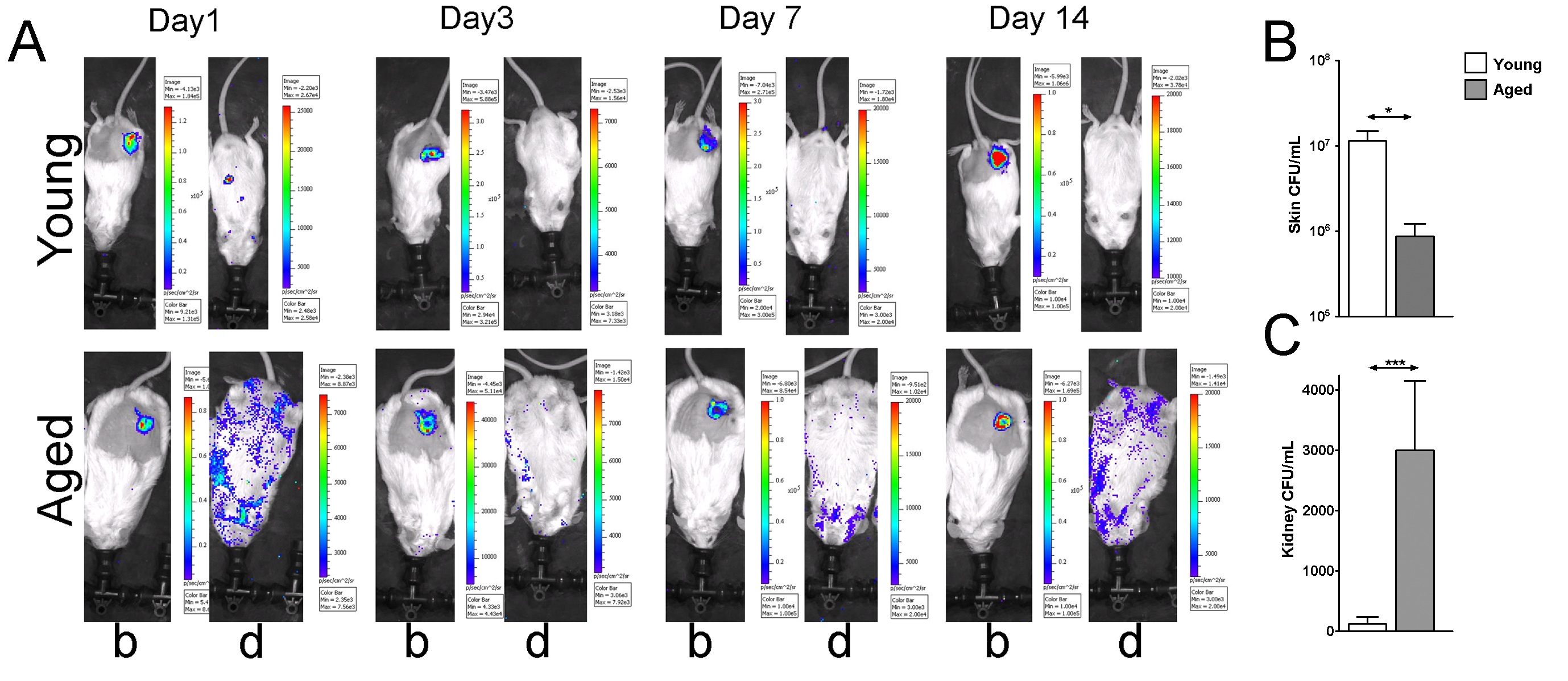

Supplement: Figure S1 — Examination of invasiveness and persistence of subcutaneously injected MRSA. Young and aged mice were infected s.c. with 109 CFUs of CST9 and followed for 14 days. (A) Visualization of MRSA dissemination. CST9 harboring Tn4001::luxABCDE KmR was used for infection of mice. Bioluminescence at the infection site and deep tissues was followed over a period of 14 days using an IVIS system. At least 3 mice in each age group were infected for each time point for IVIS analysis. b: back of mice; d: dorsa of mice. (B) Skin lesion CFU (n = 6); (C) CFU in Kidneys (n = 6). (TIF) [file pone.0041454.s001.tif]

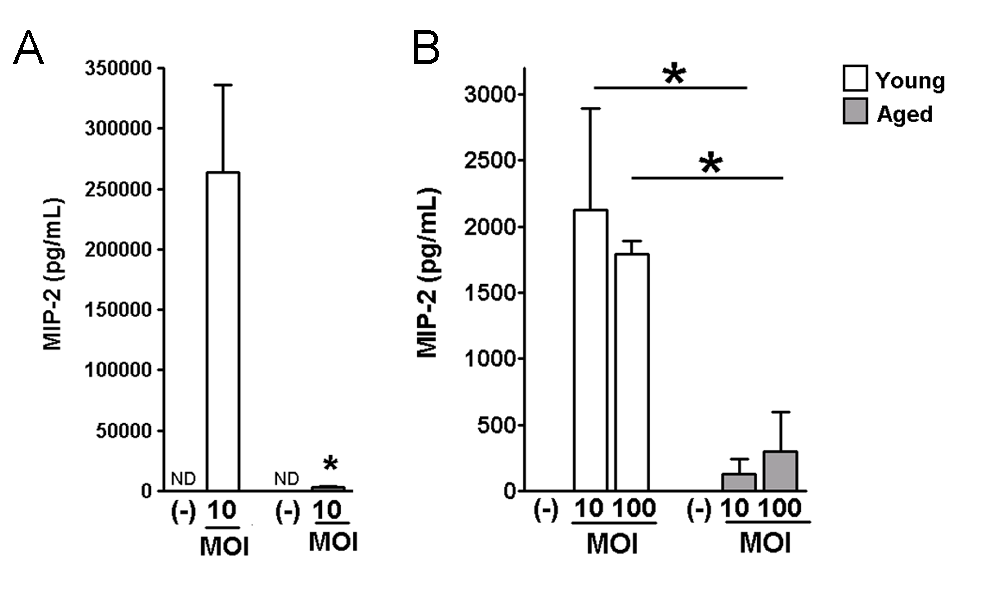

Supplement: Figure S2 — Macrophages and skin fibroblasts isolated from aged mice show reduced MIP-2 response to MRSA infection. (A) Macrophage MIP-2 at 18 h post-infection; (B) skin fibroblast MIP-2 at 18 h post-infection. *p<0.05, compared between young and aged mice. (TIF) [file pone.0041454.s002.tif]

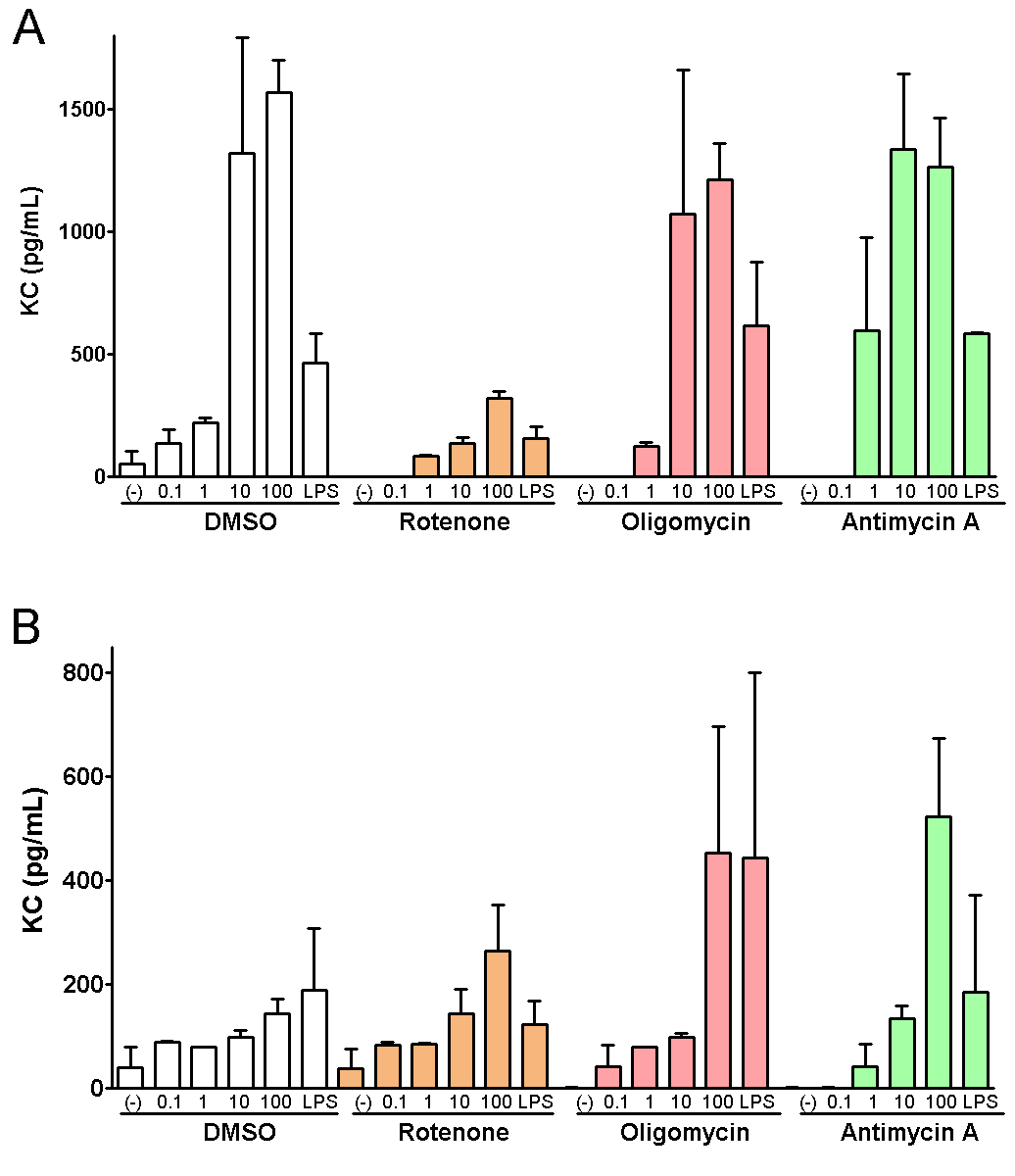

Supplement: Figure S3 — Mitochondrial electron chain complex III and V have limited effect on KC levels in skin fibroblasts isolated from both young and aged mice when responding to MRSA infection. (A) Young; (B) Aged. (TIF) [file pone.0041454.s003.tif]

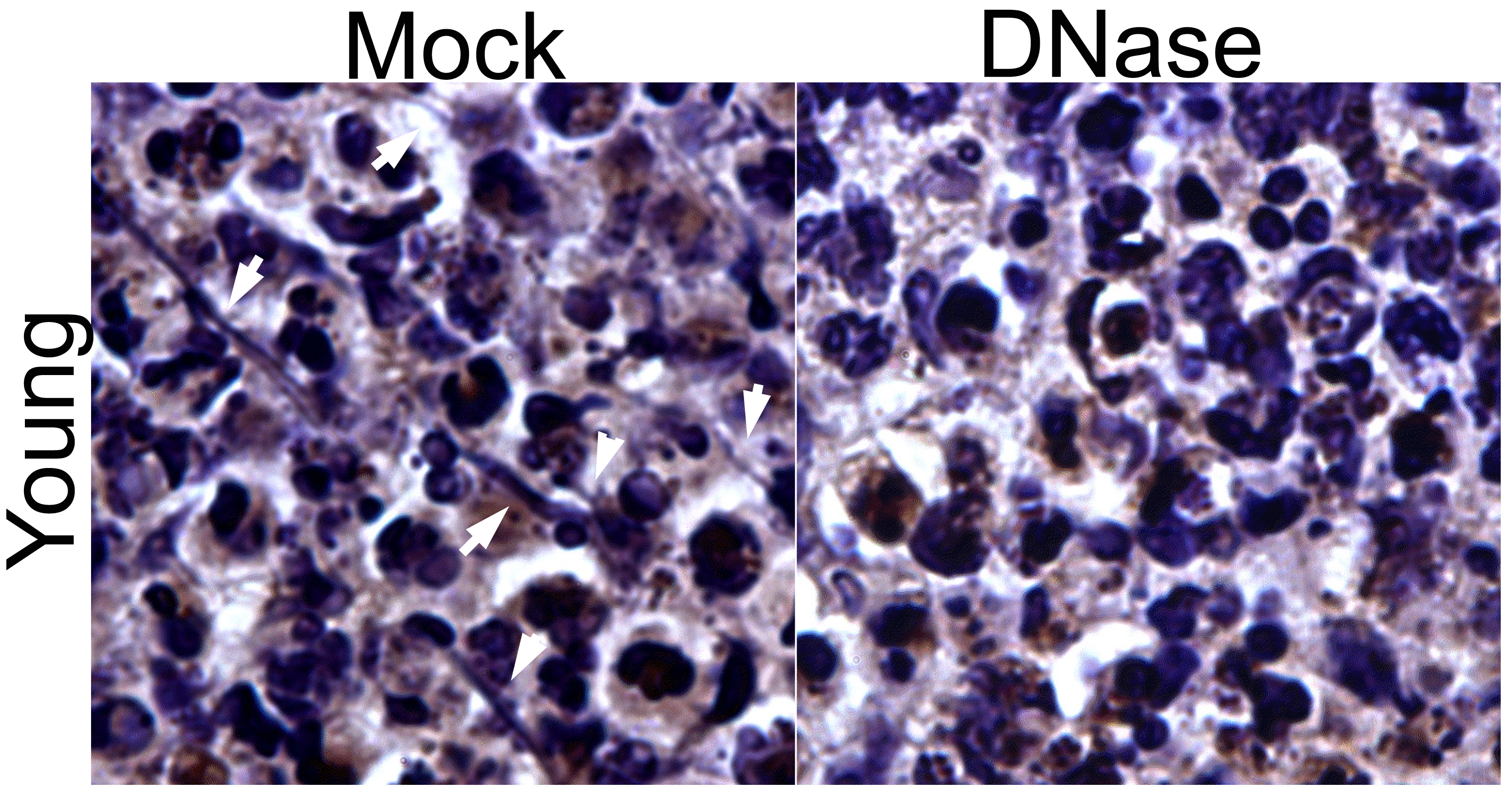

Supplement: Figure S4 — In vivo DNase administration reduced DNA fibers at the site of infection. Young mice were infected with 109 CFU of Nuc- S. aureus and half were treated daily with 1 µg DNase I. On day 3 post-infection, the mice were sacrificed and the infected tissues were excised. After embedding, the tissue slices were analyzed for the presence of elastase by immunohistochemistry. (TIF) [file pone.0041454.s004.tif]
