# Supplementary material for: Innate Immune Dysfunctions in Aged Mice Facilitate the Systemic Dissemination of Methicillin-Resistant S. aureus
Source: PLoS One. 2012 Jul 26;7(7):e41454. doi: 10.1371/journal.pone.0041454 (PMC3406035; doi:10.1371/journal.pone.0041454)
Supplement: File S1 — Supporting methods and materials. (DOC) [file pone.0041454.s005.doc]

**Supporting methods and materials**

*Visualization of murine skin infection using bioluminescent MRSA*

Isogenic CST9 strain harboring Tn*4001 luxABCDE* Km^R^ (15) was constructed using previously established transduction methods (16). Two- and 16- to 22-month-old mice were infected with approximately 10^9^ CFU of CST9 Tn*4001 luxABCDE* Km^R^ on each flank. Infection of mice was performed per protocols described in *Methods and Materials*.

The bioluminescence from the infected animals was determined using an ICCD camera of Xenogen’s LivingImage system (Xenogen Corporation).

*Inhibition of mitochondrial electron chain complex III and V*

For treatment using inhibitors of mitochondrial electron chain complexes, 1 x 10^5^ isolated skin fibroblasts were plated in each well in a 24 well plate. Cells were incubated with or without antimycin (Complex III inhibitor) 5 µg/mL, or oligomycin (Complex V inhibitor) 5 µg/mL in DMEM-5% FBS with 5% CO_2_ at 37^o^C. After 18 hours of treatment, cells were washed once with HBSS once. Cells were stained with trypan blue to verify the viability of the cells before infection of MRSA. Diluted MRSA were added to each well to a final MOI of 0.1 to 100 in DMEM-5% with or without 5 µg/mL antimycin or 5 µg/mL oligomycin. To facilitate the interaction of cells and MRSA, the tissue culture plates containing isolated cells and MRSA were centrifuged at 700 x *g* for 10 min at room temperature. After centrifugation, the cells were incubated at 37^o^C with 5% CO_2_ for 3 and 18 hours. After incubation, the culture supernatants were collected and submitted for ELISA.
